# Wood’s Medical and Surgical Monographs

**Published:** 1889-09

**Authors:** 


**Wood's Medical and Surgical Monographs, Vol. III., No. 2.** August, 1889. Contents: The Treatment of Syphilis at the Present Time. By Dr. Maximilian Von Zeissl. The Treatment of Inebriety in the Higher and Educated Classes. By James Stewart, B. A. Manual of Hypodermic Medication. By Drs. Bourneville and Bricon.

The titles in this eighth number of these valuable publications are upon subjects of importance. That they are well considered, needs no further comment than to point to the names of the authors.

It behooves every physician to make himself familiar with the latest literature upon the subject of syphilis, for it is a disease that influences and modifies almost every other malady, and is so subtle withal that the closest scrutiny is sometimes inadequate to detect it.

Hypodermic medication is constantly coming more and more to play an important part in the therapeutic management of disease. Hence we are glad to see specific literature put forth upon the subject. Much ignorance, fear, and even carelessness prevails in this field, and we are hopeful that this book will be widely read, for it cannot fail to remove many of those stumbling-blocks to progress.

